# How do German pharmacologists publish in the non-peer-reviewed science magazine *Biospektrum*?

**DOI:** 10.1007/s00210-023-02740-x

**Published:** 2023-09-30

**Authors:** Helena Zöllner, Roland Seifert

**Affiliations:** Department of Pharmacology, Hannover Medical School, Carl-Neuberg-Str. 1, D-30625 Hannover, Germany

**Keywords:** Biospektrum, *Naunyn–Schmiedeberg’s Archives of Pharmacology*, Covid pandemic, Gender studies, Pharmacology

## Abstract

Publications in peer-reviewed journals are the most important currency in science. But what about publications in non-peer-reviewed magazines? The objective of this study was to analyze the publications of scientists, with a focus on pharmacologists, in the non-peer-reviewed German science magazine *Biospektrum* from 1999 to 2021. *Biospektrum* is edited by five scientific societies in Germany including the Society for Experimental and Clinical Society Pharmacology and Toxicology (DGPT) and provides opportunities to researchers to showcase their research to a broad audience. We analyzed 3197 authors of 1326 articles. Compared to the fields of biochemistry, microbiology, and genetics, pharmacology was largely underrepresented. Just three institutions in Germany contributed most papers to *Biospektrum*. Researchers with a doctoral degree were the largest author group, followed by researchers with a habilitation degree. Among all major fields, women were underrepresented as authors, particularly as senior authors. The Covid pandemic leads to a drop of publications of female first authors but not last authors. Compared to publications in the peer-reviewed journal *Naunyn–Schmiedeberg’s Archives of Pharmacology* (Zehetbauer et al., Naunyn-Schmiedebergs Arch Pharmacol 395:39–50 (2022)), female pharmacologists were underrepresented in the *Biospektrum*. Thus, German pharmacologists as a group do not value investing in “social impact” gained by publications in *Biospektrum*, and this attitude is even more prominent among female pharmacologists. Investing less in “social impact” by female pharmacologists may result in reduced visibility on the academic job market and may contribute to reduced opportunities to achieve high academic positions.

## Introduction

Publications in peer-reviewed journals are the most important currency is science, determining the academic career success of individual scientists (Sharma et al. 2014). Several gender inequalities for various aspects of academic life, including editor positions, peer review, and first and senior authorships, have been noted across multiple disciplines (Roper 2019; Segovia-Saiz et al. 2020; Pinho-Gomes et al. 2021; Balasubramanian et al. 2020; González-Alvarez and Sos-Pena 2020; Hagan et al. 2020; Bagga et al. 2021; Bram et al. 2022; Squazzoni et al. 2021; Helmer et al. 2017; Budden et al. 2008). In a recent study, we examined the contributions of female pharmacologists from Germany to *Naunyn–Schmiedeberg’s Archives of Pharmacology* from 2000 to 2020 (Zehetbauer et al. 2022). Women were underrepresented as first authors and particularly as senior authors and editors.

In marked contrast to the analysis of peer-reviewed journals, data on publication behavior of scientists in non-peer-reviewed scientific magazines is scant. The aim of this work, therefore, was to determine the publication behavior of German scientists, with a focus on pharmacologists, in the journal *Biospektrum* (https://www.biospektrum.de) from 1999 to 2021. The magazine is published by Springer Nature in cooperation with the Association for General and Applied Microbiology (VAAM), the Society for Genetics (GfG), the Society for Biochemistry and Molecular Biology (GBM), the German Society for Experimental and Clinical Pharmacology and Toxicology (DGPT), and the Association of Biology, Life Sciences and Biomedicine (VBio) and is not peer-reviewed. The above-named scientific societies support publication of the journal financially via the membership fees. In return, each member of the societies receives a print edition of each issue of the journal. Overall, the journal reaches almost 17,000 members of scientific societies in Germany. Since many of the articles in *Biospektrum* are open access and are available online, the magazine radiates far beyond the society members. In 2022, *Biospektrum* received more than 45,000 article downloads, pointing to a broad recognition and readership. The journal has 7 issues per year. It provides a forum for scientists to showcase their research in the form of mini reviews published in German language with an abstract in English. While an author cannot earn “impact factor points” when publishing in *Biospektrum*, an author can gain “social impact points” within the German research community, rendering him/her more known and, potentially, more competitive for higher academic positions. For a gender analysis of authors, *Biospektrum* is particularly suited because each article is accompanied by a photo of the researcher/research group and biosketches providing details of the academic career. In peer-reviewed journals, such information is not provided.

## Materials and methods

To investigate these developments, the electronic journal library of the Hannover Medical School and the online archive of *Biospektrum*, which offers online open access to the journal, were used. Depending on the year of publication, the data were collected in a certain way. The years 1999 to 2010 were recorded using the online archive of *Biospektrum*, while the electronic journal library of the Hannover Medical School was used for the years 2011 to 2021. The collected data were then transferred to an excel pivot table, which, thanks to its programming, already allowed brief conclusions to be drawn about the development of the recorded datasets, while still collecting data (Table 1). In this table, the articles were sorted according to various criteria. First, the title of each article was listed, which was then assigned to a scientific specialty (scientific field). This was followed by the first, second, third, fourth, and fifth author, whose gender was determined by their first name and photo in the article. The academic degree of each author was then listed. This information was retrieved from the biosketches attached to each article. After this, the issue number of the journal, the year in which the article was published, the beginning and end page of each article, and the volume of the journal were registered.

**Table 1:** Data collection in Excel

Title	Specialty	Male author 1	Female author 1	Academic degree 1	Male author 2	Female author 2	Academic degree 2	Issue number	Page no 1	Last Page no	Year
xxx	xxx	1	0	Student	0	1	Professor	2	70	72	2006
xxx	xxx	0	1	PhD	1	0	Professor	2	73	75	2006
xxx	xxx	0	1	PhD	1	0	Professor	3	80	87	2007
xxx	xxx	1	0	Student	0	1	Professor	3	88	90	2007

For better visualization, the original Excel table was translated into English, summarized, and transferred into word. This table is for visualization purpose only and should only be considered to understand the data collection. The given examples are not part of the real dataset for personal data protection reasons

Due to the large time span analyzed, changes regarding the format of the articles of the *Biospektrum* took place and impacted the data collection. For the years 2007 to 2021, the “science” part and the “special” part were included in the data collection. From the year 2002 to 2006 also the chapter “spotlight” and in the most previous recorded years, being 1999 to 2001, the chapter “overview” was included into data analysis. A total of 1327 articles with 3197 authors from 39 scientific specialities were recorded this way. All issues of *Biospektrum* from 1999 to 2021 were included.

Due to the programming of the used Excel table, different filters (e.g., specialty, academic degree, issue number, year) could be applied, and the result was obtained right away (Table 2). In the next step, these results were then visualized using GraphPad Prism version 9.

**Table 2:**
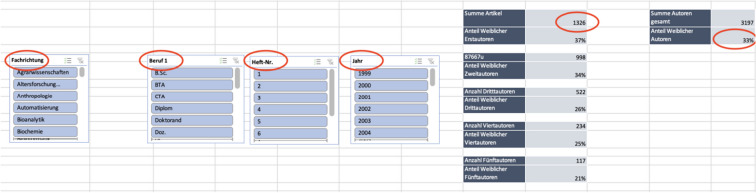
Data analysis in Excel

Visualization of different filters to analyze the collected data

## Results

**Fig. 1:**
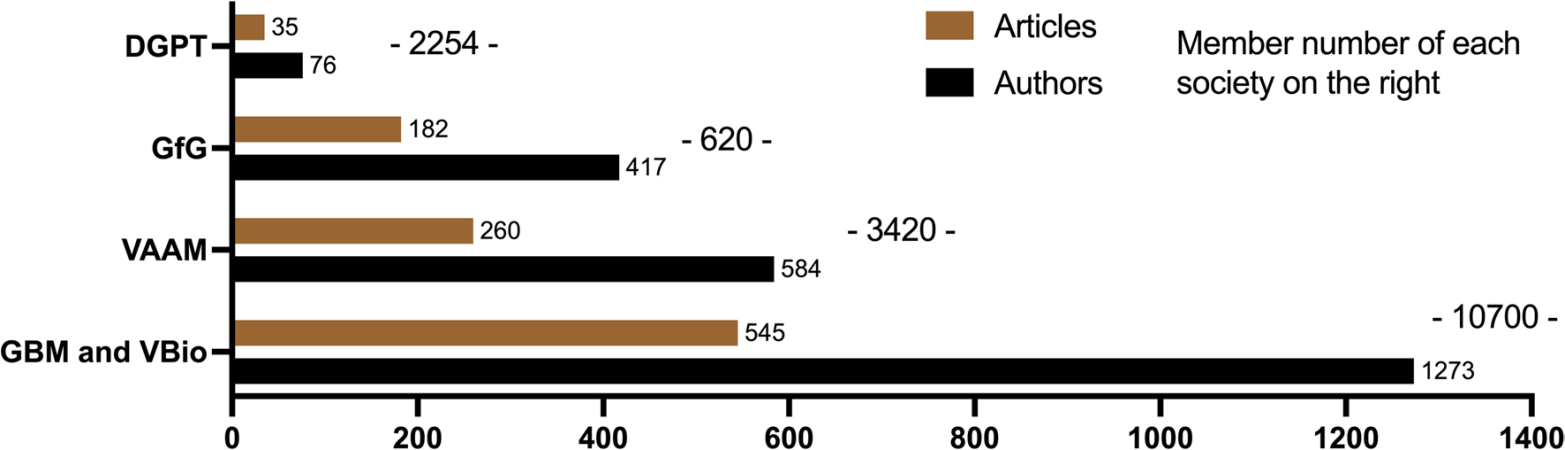
Professional societies publishing in *Biospektrum.* Publication extents of the Society of Biochemistry and Molecular Biology (GBM); the Association of Biology, Biosciences, and Biomedicine Germany (VBio); the Association of General and Applied Microbiology (VAAM); the Society of Genetics (GfG); and the German Society for Experimental and Clinical Pharmacology and Toxicology (DGPT) in *Biospektrum*

Overall, *Biospektrum* represents five professional societies with close to 17,000 members (Fig. 1). The members of GBM and VBio were pooled because the scientific fields overlap. These two societies have the largest share of members, followed by VAAM, DGPT, and GfG. The articles published in *Biospektrum* were assigned to the scientific fields represented by these five societies. The scientific societies publish at different extents in *Biospektrum*. Overall, 545 articles by 1273 authors were assigned to GBM, which has a female count of 41.3% (05/06/2023), and VBio, while 260 articles by 584 authors were assigned to VAAM with a female proportion of 46.6% (05/06/2023). In aggregate, 182 articles by 417 authors were assigned to GfG, while the DGPT, which has 40% female members (2021), only published 35 articles by 76 authors. In average, each member of GfG contributed 0.30 papers to *Biospektrum*, each member of VAAM 0.08 papers, each member of GBM and VBio 0.05 papers, whereas the DGPT contributed only 0.016 papers per member. Based on membership numbers, 17% of all VAAM members contributed to *Biospektrum*. The corresponding numbers for GfG were 67%, for GBM and VBio 12%, and for DGPT just 3.3%.

**Fig. 2:**
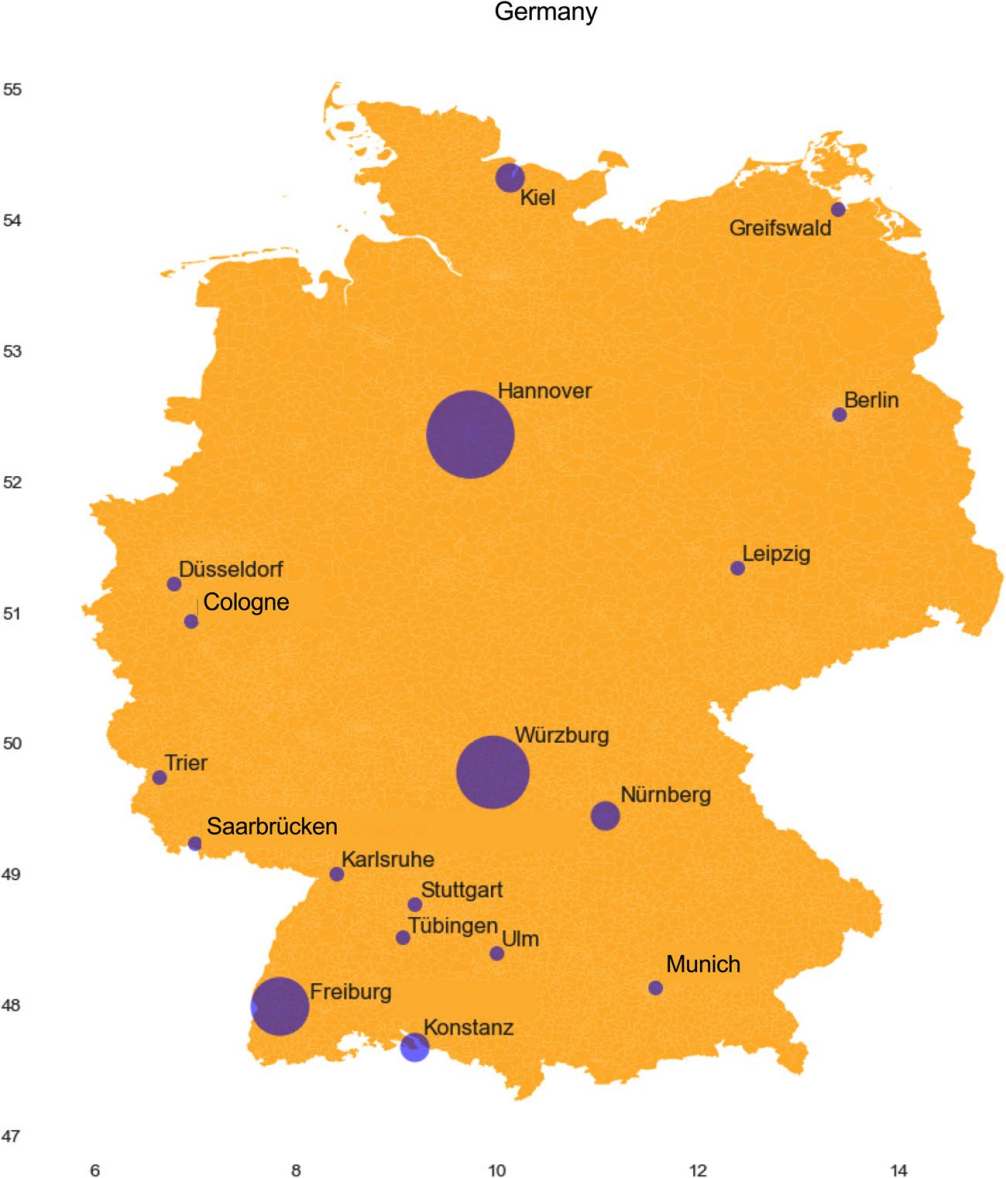
Geographical distribution of pharmacological publications in *Biospektrum*. The size of the city symbols reflects the number of papers published in *Biospektrum*

Out of the 35 papers published by the DGPT, just three universities contributed the largest share of publications in pharmacology in Germany (Fig. 2). The Hannover Medical School was first with 6 papers, followed by the University of Würzburg (5 papers), and the University of Freiburg (4 papers)*.* Other universities contributed just one or two papers, and many universities did not contribute to *Biospektrum* at all.

**Fig. 3:**
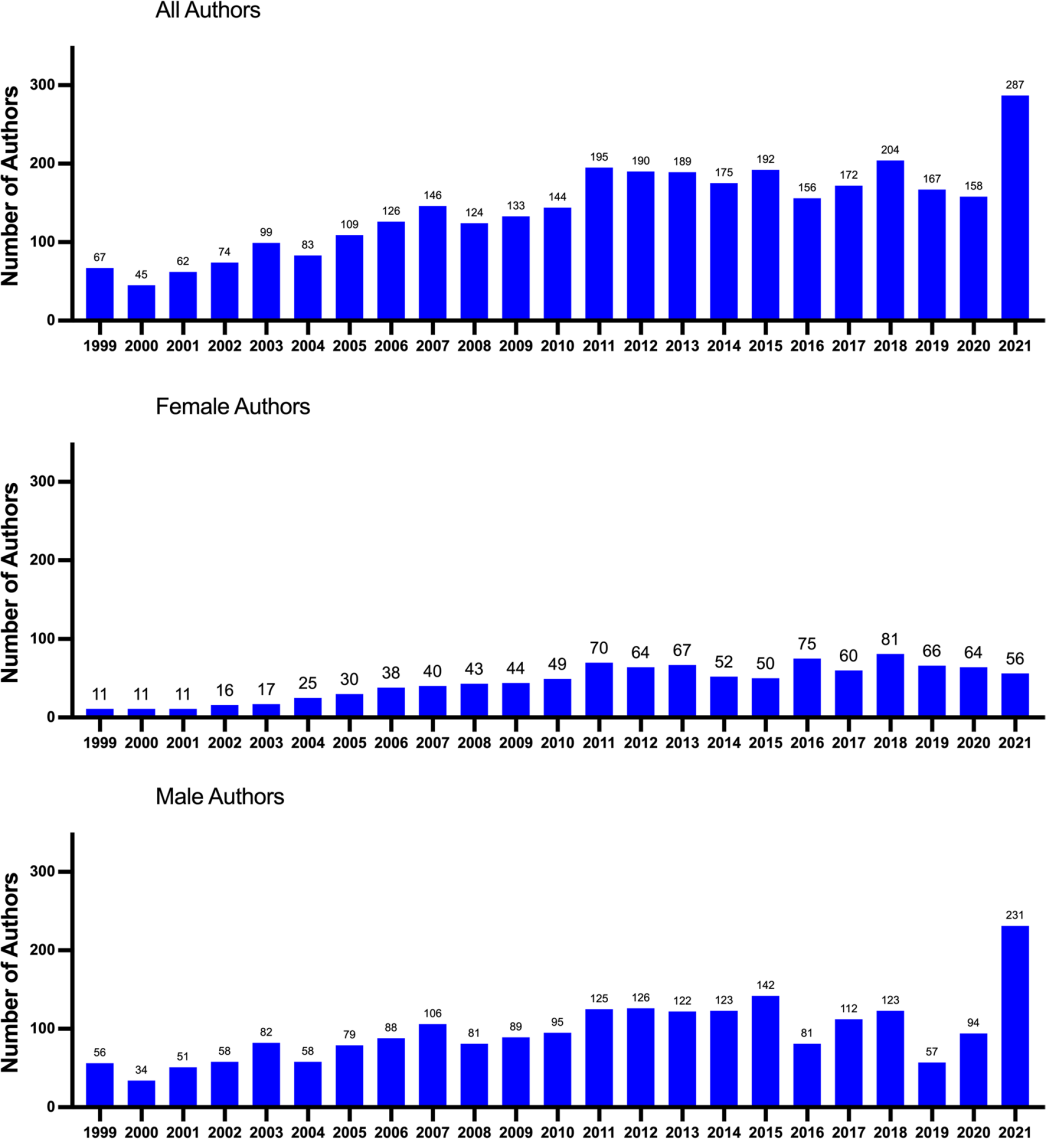
Number of authors in all collected articles of *Biospektrum*. The total number of authors and the numbers of male and female authors from 1999 to 2021 are listed

The overall number of authors in *Biospektrum* increased from about 50 authors per year in the first 3 years of its existence to a plateau between 160 and 200 authors per year between 2011 and 2020 (Fig. 3), indicating the increasing importance and recognition of *Biospektrum* in the biomedical sciences in Germany. In 2021, an exceptionally large number of authors (close to 270) were noted. Starting with just 11 female authors in 1999, a growth was apparent within the number of female authors reaching a broad plateau with 50–80 authors per year between 2011 and 2021. The number of male authors started with 56 in 1999. In contrast to the female authors, the number of male authors showed large variation over the time period analyzed with an exceptional peak of 231 authors in 2021. In most years, the number of male authors surpassed the number of female authors.

Table 3 provides an overview on the number of authors and the gender distribution among different scientific specialties. Overall, 3176 authors were counted, with 2133 male authors (67.14%) and 1043 female authors (32.86%). The top subject covered in *Biospektrum* was microbiology with a female share of 36%. Biochemistry, biology, and molecular biology were also strongly represented in *Biospektrum* with a share of 33–35% of female authors. The top 10 topics in *Biospektrum* were completed (in descending order) by biotechnology, medicine, cell biology, chemistry, molecular medicine, and pharmacology/toxicology. Pharmacology/toxicology was represented by just 76 authors (2.39% of all authors) although the DGPT contributes 13.6% of all members (see Fig. 1) of the societies represented in *Biospektrum*. Among the top 10 specialties, representing a total of 2850 authors (89.67% of all authors publishing in *Biospektrum*), pharmacology/toxicology by far had the lowest female author proportion, lagging more than 10 percent points behind the specialty with the second-lowest female percentage (chemistry). Even when the top 15 specialties were considered, pharmacology/toxicology had the lowest rank in female author share (together with physics). For specialties with author numbers < 20, reliable assessment of female/male author shares became arbitrary and, therefore, was not performed.

**Table 3:**
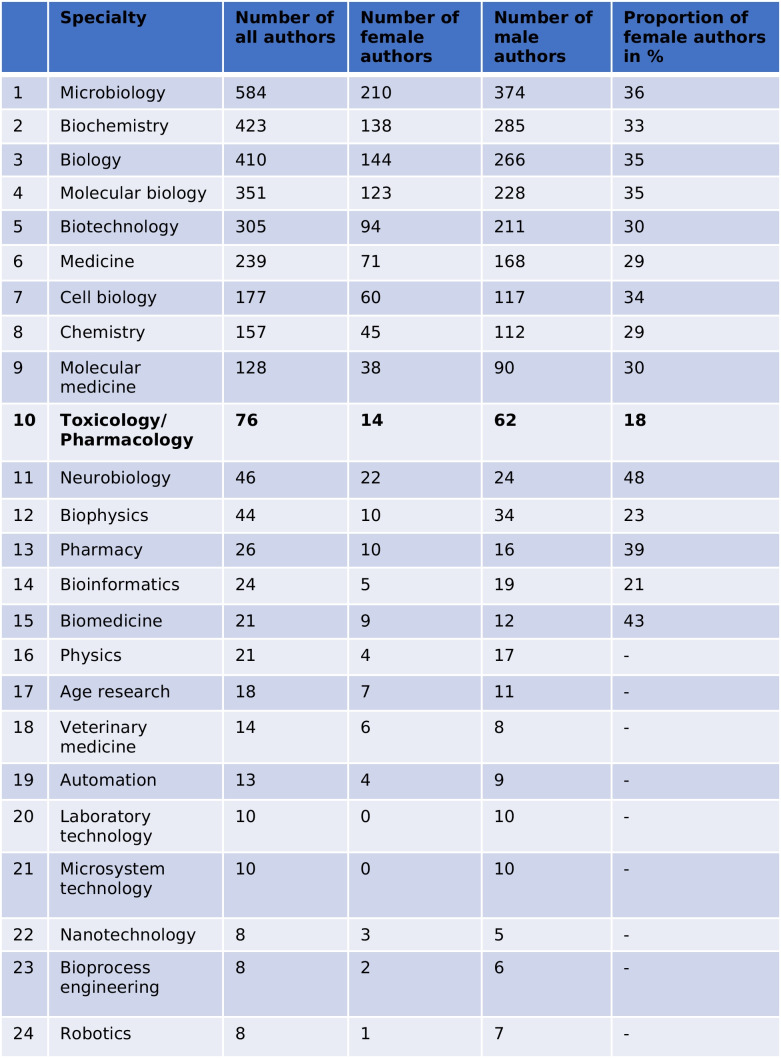

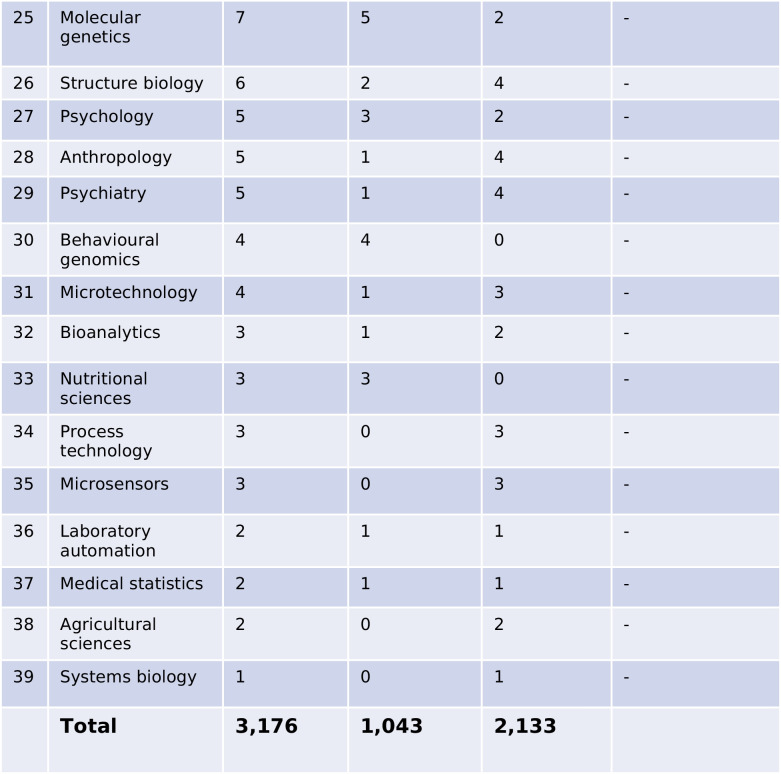
Scientific specialties and the gender proportion of authors

The numbers for toxicology/pharmacology and the total numbers are particularly important for the analysis and are, therefore, highlighted in bold

In the biomedical sciences in general and in Germany in particular, it is a long tradition (“an unwritten law”) that the first author is the person who performed most of the work (experiments and writing for original papers and writing for review papers such as for *Biospektrum*) (Bhattacharya 2010; Zehetbauer et al. 2022). Usually, it is a scientist with a doctoral degree (“postdoc”). In general, the last author is a senior scientist heading the research group with a habilitation degree or a professor position.

**Fig. 4:**
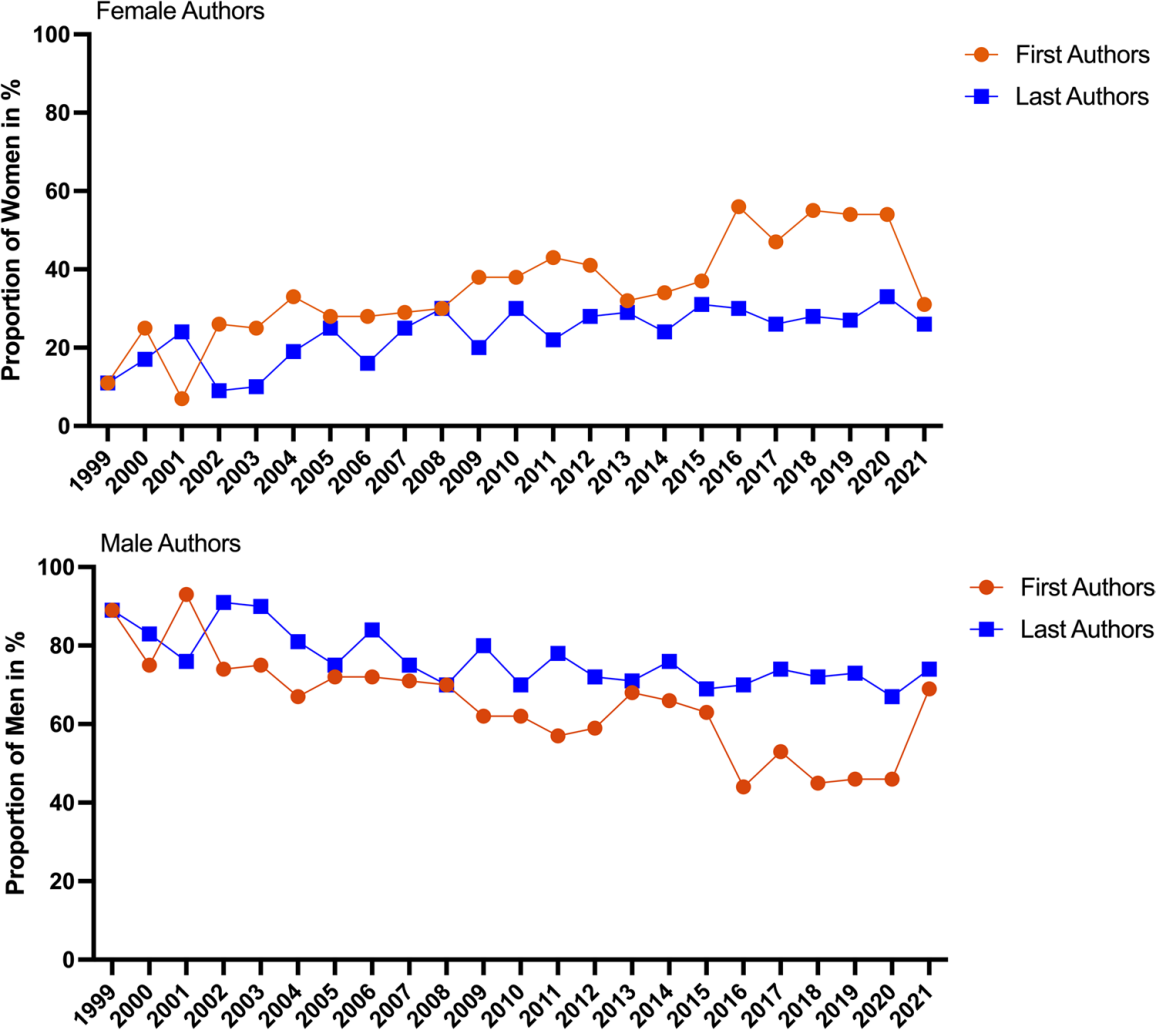
Proportion of female and male authors in *Biospektrum* from 1999 to 2021. First authors and last authors are listed. Relative numbers (percentages) are presented

Starting with just four female first authors in 1999, a peak with 35 female first authors in *Biospektrum* was reached in 2011 (absolute numbers are not shown; percentages are shown in Fig. 4). A second peak was reached in 2018 with 41 authors. The number of female first authors dropped sharply from 2020 to 2021. The number of male first authors showed substantial fluctuation over the time period analyzed and showed a sharp increase from 2020 to 2021. The number of female last authors increased steady over the years and reached a stable plateau between 2016 and 2021 with around 20 authors. The number of male last authors also increased over the years and reached a plateau at around 50 authors between 2012 and 2021. Thus, among senior authors in *Biospektrum*, men were represented at an about 2.5-fold higher number than women.

When expressed in percentage, an increase in female first authorship from 1999 (about 10%) to about 50% (2015–2020) was recorded, with a sharp drop in 2021. An increase in female last authorship from about 10% in 1999 to about 25–30% between 2010 and 2021 was recorded. For males, a continuous decrease in first authorship was evident from 1999 to 2020, with a sharp increase in 2021. For males, the proportion of senior authorships declined from about 90% in 1999 to a broad plateau of around 70–75% between 2010 and 2021.

**Fig. 5:**
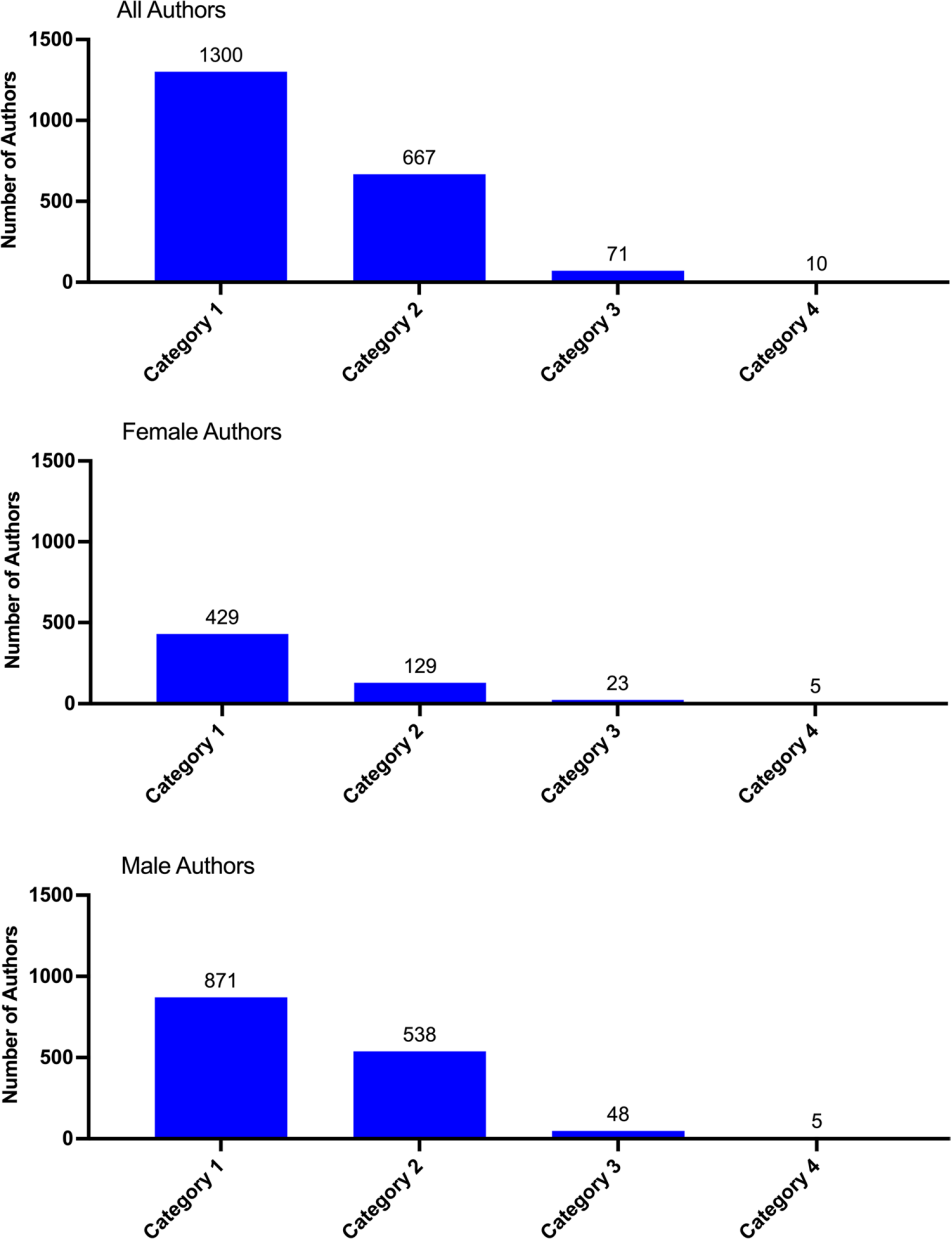
Categories of authors in *Biospektrum* from 1999 to 2021. Academic degrees of authors are listed for all authors, and female and male authors in descending order. Category 1, doctoral degree; category 2, private lecturer (habilitation), professor. Category 3, diploma, master of science; category 4, technical assistant

Figure 5 analyzes the authors in *Biospektrum* according to their academic degree. Researchers with a doctoral degree constituted the largest group of *Biospektrum* authors (category 1), followed by authors with a habilitation degree or professor position (category 2). Authors with a diploma degree or master of science degree (category 3) or professional technicians (medical assistants (MTA), chemical assistants (CTA) and biological assistants (BTA)) (category 4) played only a minor role as *Biospektrum* authors. Among female authors, category 2 researchers accounted for 23% of all female authors with a higher academic degree (category 1 + 2), whereas for male authors, category 2 researchers accounted to 38% of all male authors with a higher academic degree (category 1 + 2). In category 1, females were represented at 49% of the male number, but in category 2 only at 24% of the male number.

## Discussion

*Biospektrum* is a unique non-peer-reviewed scientific magazine. It is edited by a globally leading publisher (Springer Nature), is edited on behalf of five leading biomedical research societies in Germany, has a broad resonance room among society members and beyond, and provides a unique opportunity for biomedical scientists to showcase their research. The magazine is also unique in the sense at it provides short biosketches and photos of the authors, facilitating assignment of gender and specific academic degrees to authors. We are not aware of another non-peer-reviewed scientific magazine that is being edited for five different scientific societies.

Usually, *ASBMB Today* (https://www.asbmb.org/asbmb-today) or *Pharmakon* (https://www.dphg.de) being prime examples, non-peer-reviewed scientific magazines are edited on behalf of just one scientific society. Thus, *Biospektrum* offers an excellent opportunity to analyze cultural differences of scientific publishing in non-peer-reviewed magazines.

One may argue that publications in such magazines are not of much value because no impact factor points, critical for a successful academic career, are being accrued. However, non-peer-reviewed magazines offer a unique opportunity to showcase a scientist’s research program and to get known within the research community, both scientifically, visually (a photo of the scientist is published) and career-wise (a biosketch is published). This “social capital” gained by a publication in *Biospektrum* may contribute to securing higher academic positions in the future. It should also be noted that articles in *Biospektrum* are much shorter than classic reviews in peer-reviewed journals and published in German language, i.e., the effort and time required to put together an article for *Biospektrum* are much lower than for a peer-reviewed journal.

Strikingly, there are substantial cultural differences among scientific societies in terms of valuing publications in *Biospektrum*. The GfG, smallest scientific society represented in *Biospektrum*, made the largest scientific contribution to the magazine relative to its membership number. Geneticists appear to value a publication in *Biospektrum* very highly. In contrast, the DGPT, a medium-sized scientific society, comparable in size to VAAM, values publications in *Biospektrum* much less than GfG and VAAM. Thus, there are substantial cultural differences among scientific societies in Germany in terms of using *Biospektrum* as forum for spreading scientific ideas and showcase research achievements. To our knowledge, there are no published data on this topic yet to put our present findings into a broader context.

Showcasing research accomplishments is particularly important for scientists who have not yet secured a permanent academic position, usually a tenured professor position. This group of scientists has a doctoral degree and is typically employed in temporary academic positions, ranging from few months to 5 years. Fitting to these professional career requirements, the largest author group in *Biospektrum* are scientists with a doctoral degree. They use *Biospektrum* as a platform to promote their research and accumulate “social capital.” And over the years of its existence, *Biospektrum* has become more and more popular as evidenced by the increasing number of authors and the high article download numbers. Typically, postdoctoral researchers are in their earlier thirties, an age where there is dual requirement for promoting the professional career and establishing an own family. It has been amply documented that the Covid pandemic had negative impact on productivity, particularly for female researchers with small children, i.e., female researchers spent much less time on research and much more time on childcare because childcare facilities and schools were closed. It was noted repeatedly that male researchers were much less affected by the Covid pandemic because they spent much less time on childcare (Caldarulo et al. 2022; Jasrotia and Meena 2021; Myers et al. 2020). These facts are supported by the present study. Particularly, in 2021 (the second year of the pandemic), a strong decrease in female first authorships and a massive increase in male first authorships in *Biospektrum* was noted, i.e., the productivity of female doctoral degree holders substantially declined whereas the productively of male doctoral degree holders not only remained stable but even increased (Fig. 4).

The DGPT uses the *Biospektrum* much less intensively than other professional societies. These papers show a clear geographical distribution: Even though there are many pharmacological institutes performing excellent research in Germany, only very few of them are responsible for publications in *Biospektrum*. It is apparent that just in three pharmacological institutes in Germany, publications in *Biospektrum* are valued very much (Fig. 2), whereas in other pharmacological institutes, being very productive in terms of research (Zehetbauer et al. 2022), publications in *Biospektrum* are not valued very much. For example, Heidelberg and Bonn are not represented in *Biospektrum*. Thus, in addition to a discipline-wide attitude toward publishing in *Biospektrum*, there are striking local hotspots for pharmacological publications in *Biospektrum*. The most likely explanation for the discrepancies between overall research productivity of pharmacology institutes in Germany in terms of papers in peer-reviewed journals and their very modest presence in *Biospektrum* is the attitude of the respective institute directors (professors) toward the magazine.

The present study also unmasked unexpected gender aspects of publication behavior in non-peer-reviewed magazines. Overall, over the past 25 years, the proportion of females as first and last authors in *Biospektrum* increased, but since about 2010, the academic system appears to be rather stable in terms of gender proportion, despite substantial political measures for gender equality in science. Similar observations were recently made for publications in a peer-reviewed pharmacological journal (Zehetbauer et al. 2022). In most biomedical research fields covered in *Biospektrum*, the proportion of female authors ranges between 30 and 40%, fitting quite well to the proportion of females in GBM, GFG, and VAAM. Strikingly, in pharmacology, the female proportion among *Biospektrum* authors was just 18% although 40% of all DGPT members are female. Among all major biomedical fields analyzed in *Biospektrum*, pharmacology ranked very low.

What could be the reason for this striking underrepresentation of female pharmacologists among authors in *Biospektrum*? Since there is no peer review of pharmacological papers in *Biospektrum* and every submitted paper is ultimately published, we can exclude some hidden paper selection bias as reason for the female underrepresentation. Rather, the executive board of the DGPT has always encouraged submissions to *Biospektrum*. In other words, *Biospektrum* is a glass ceiling–free journal for female pharmacologists. We can also exclude that writing for *Biospektrum* constitutes an excessive work burden because the articles are short and in German language, the mother tongue of most pharmacologists in Germany. Last but not least, the layout of the finally published papers is very appealing.

So why is publishing in *Biospektrum* for female pharmacologists so unattractive although the “social capital” benefits should be evident? In a recent study, Zehetbauer et al. (2022) analyzed the peer-reviewed publications of female pharmacologists from Germany in *Naunyn–Schmiedeberg’s Arch Pharmacol*. In terms of geography, time period analyzed, and number of authors analyzed, the latter analysis is comparable with the present analysis of *Biospektrum* (Table 4). It is apparent that overall, females are substantially underrepresented compared to males in terms of authorship in the peer-reviewed journal, but the representation of women in *Naunyn–Schmiedeberg’s Arch Pharmacol* (28% of all authors) is closer to the representation of authors from all biomedical disciplines in *Biospektrum* (33%). It is very unlikely that the work burden in pharmacology is much greater than in other biomedical field to secure a publication in a peer-reviewed journal because the methodological approaches in research are rather similar across fields these days. Accordingly, different time availability across fields is unlikely to explain differences in publication behavior in *Biospektrum*. Thus, we must assume that female pharmacologists make conscious or unconscious decisions for not publishing in *Biospektrum*. Apparently, they do not see the added value in publishing in this magazine. Most likely, this is the result of a cultural attitude within the discipline that pharmacology institute directors do not point out sufficiently to their postdoctoral researchers the importance of showcasing their research in *Biospektrum*. Thus, female pharmacologists invest substantially in their “impact factor portfolio” (Zehetbauer et al. 2022) but neglect their “social impact portfolio.” Female authors in pharmacology seem to have an economically driven point of view (investment in “real” impact factor points) and do not invest into the “social capital” that is gained when publishing in *Biospektrum*. The fact that scientists who publish in this magazine are better known in the German biomedical research community, even without peer-review, seems to be better known to male pharmacologists when compared to their female colleagues.

**Table 4:** Comparison the studies of Zehetbauer et al. (2022) with the present study

Zehetbauer et al. (2022)	Present study
Analyzed years: 20 (2000–2020)	Analyzed years: 22 (1999–2021)
Country analyzed: Germany	Country analyzed: Germany
Number of specialties analyzed: 1 (Pharmacology)	Number of specialties analyzed: 39 specialties
2886 authors - 2071 men (72%) - 815 women (28%)	3197 authors - 2147 men (67%) - 1050 women (33%) Pharmacology - 62 men (82%) - 14 women (18%)

Comparison of the study performed by Zehetbauer et al. (2022) and of Zöllner and Seifert (2023) illustrating the proportion of female authors within pharmacology across the years 1999 to 2021

This study has three important methodological limitations. Firstly, membership in one of the five scientific societies is not a conditio sine qua non for publishing in *Biospektrum*. Secondly, in a published article in Biospektrum, membership of a given author in a scientific society is not mentioned. Thirdly, a given article in *Biospektrum* may often be assigned to multiple specialties. Thus, there was some “educated guessing” how to assign a paper to the “best fitting” scientific specialty and scientific society. However, the authors of this paper are very familiar with the field of pharmacology so that for these papers, assigning bias was minimal. Being aware of these limitations, we focused on the peculiarities of publishing behavior in pharmacology relative to all other biomedical fields and not so much on differences among other fields. This will be the task of scientists who are more intimately familiar with the fields of microbiology, biochemistry, molecular genetics, and cell biology.

In conclusion, the present analysis of *Biospektrum*, a leading non-peer-reviewed science magazine in Germany, confirms the notion that women are underrepresented as authors in biomedical sciences. The Covid pandemic negatively affected the publication output of junior female scientists both in peer-reviewed and non-peer-reviewed journals. We also uncovered substantial cultural differences among scientific disciplines to use *Biospektrum* as publication outlet. Pharmacology is a massively underrepresented field, and female scientists in this field invest much less effort than their male colleagues in publishing in a non-peer-reviewed journal. Rather, women focus on publishing in peer-reviewed pharmacological journals. But the price for this decision may be that female pharmacologists accumulate less “social capital” than men which then reduces their chances to secure higher academic positions. In search committees for professor positions in pharmacology, there are typically not only pharmacologists represented but also scientists from other biomedical fields who read *Biospektrum*. Future studies will have to analyze whether authors publishing in *Biospektrum* as postdocs in the position as first authors will have increased chances of securing prominent permanent academic positions.

## Data Availability

All source data for this study are available upon reasonable request.

## Abbreviations

DGPT: German Society for Experimental and Clinical Pharmacology and Toxicology (Deutsche Gesellschaft für Experimentelle und Klinische Pharmakologie und Toxikologie)
GBM: Society of Biochemistry and Molecular Biology (Gesellschaft für Biochemie und Molekularbiologie)
GfG: Society of Genetics (Gesellschaft für Genetik)
VAAM: Association of General and Applied Microbiology (Vereinigung für Allgemeine und Angewandte Mikrobiologie)
VBio: Association of Biology, Biosciences, and Biomedicine Germany (Verband Biologie, Biowissenschaften und Biomedizin in Deutschland e.V.)
